# Wearable Devices and Nurses’ Health: Protocol for an Integrative Review

**DOI:** 10.2196/48178

**Published:** 2023-07-21

**Authors:** Susan W Buchholz, Fabrice I Mowbray, Gabrielle Allman, John P Verboncoeur, Lauren Beam, Leigh Small

**Affiliations:** 1 College of Nursing Michigan State University East Lansing, MI United States; 2 College of Engineering Michigan State University East Lansing, MI United States

**Keywords:** nurse, wearable, wearable device, mHealth, mobile health, health technology adoption and use, health outcome, adoption, usage, health technology, digital health, integrative review, literature review

## Abstract

**Background:**

Nurses comprise over half of the global health care workforce, and the nursing care they provide is critical for the global population's health. High patient volumes and increased medical complexity have increased the workload and stress of nurses. As a result, the health of nurses is often negatively impacted. Wearables are used within the health care setting to assess patient outcomes; however, efforts to synthesize the use of wearable devices focusing on nurses’ health are limited.

**Objective:**

The primary objective of our integrative review is to synthesize available data concerning the utility of wearable devices for evaluating or improving (or both) the health of nurses.

**Methods:**

We are conducting an integrative review synthesizing data specific to wearable devices and nurses’ health. The research question for this review aims to answer how wearable devices are used to evaluate health outcomes among nurses. We searched the following electronic databases from inception until July 2022: PubMed, Embase, CINAHL, Web of Science, IEEE Explore, and AS&T. Titles and abstracts were imported into Covidence software, where citations were screened and duplicates removed. Title and abstract screening has been completed; however, full-text screening has not been started. Further screening is being conducted independently and in duplicate by 2 teams of 2 reviewers each. These reviewers will extract data independently.

**Results:**

Search strategies have been developed, and data were extracted from 6 databases. After the removal of duplicates, we collected 8603 studies for title and abstract screening. Two independent reviewers conducted the title and abstract review, and after resolving conflicts, 277 full-text articles are available for review to determine whether they meet the inclusion criteria.

**Conclusions:**

This integrative review will provide synthesized data to inform nurses and other stakeholders about the extent of wearable device–related work done with nurses and provide direction for future research.

**International Registered Report Identifier (IRRID):**

DERR1-10.2196/48178

## Introduction

Globally, nurses constitute the largest body of regulated health care professionals and account for the greatest proportion of direct patient care hours [1-3]. Patient volumes and medical complexity have increased due to organizational factors (eg, understaffing) and population aging, increasing the workload and stress of nurses—these are 2 factors known to negatively influence their overall health, quality of life, and job satisfaction [4-6]. In addition, the physical and psychosocial demands of nursing have resulted in many nurses leaving direct patient care or the profession, increasing the burden of care on an already strained profession [7-9]. The negative consequences on nursing staff are reported to have a spillover effect on patient outcomes, and high-stress environments are known to negatively influence the quality of care by nurses [4,10].

The use of wearable devices is growing among the general public [11]. Wearable devices are small electronic devices that are noninvasive and are attached to a piece of clothing that a person is wearing, worn as a device anchored directly to the body, or attached directly to the skin, and have embedded sensors with wireless mobility (eg, Fitbit tracker and Apple Watch) [12-14]. There is growing interest in the use of wearables in the health care setting to provide data for monitoring and to improve the delivery of care for patients in both outpatient and inpatient settings [15-18].

Another potential application of wearable devices in the health care setting is with the staff providing patient care, including the nursing staff. Some nursing staff are already using wearable devices for personal or work-related reasons, such as physical activity monitoring [19], highlighting the feasibility of collecting wearable data and assessing its utility in informing decision-making about nurses' health. However, efforts to synthesize wearable devices that focus on nurses’ health are limited, with previous reviews focused heavily on sleep health [20,21] or those not specific to nursing [22]. Given the increasing popularity of wearables, coupled with the health challenges that nurses are currently facing, conducting a rigorous review of how wearables are being used to assess the health of nurses would provide evidence-based knowledge to inform how to move forward with the use of wearables to address the health of nurses.

Therefore, the primary objective of our integrative review is to synthesize available data concerning the utility of wearable devices for evaluating or improving (or both) the health of nurses. Specifically, we seek to evaluate the frequency of their use and the associations between wearable devices and health outcomes (physical or psychosocial) among nurses. We hypothesize that the current body of literature is suitable for synthesis, though outcomes and devices are heterogeneous.

## Methods

### Overview

We aim to conduct an integrative review synthesizing all data specific to wearable devices and the health of nurses. We used the PRISMA-P (Preferred Reporting Items for Systematic Reviews and Meta-Analyses Protocols) checklist to guide the reporting of this protocol [23]. For the final manuscript, we will use the PRISMA (Preferred Reporting Items for Systematic Reviews and Meta-Analyses) guidelines to guide the reporting of the review [24].

### Research Question

The research question for this review aims to determine how wearable devices are used to evaluate health outcomes among nurses. A PICO (population, intervention, control, and outcomes) format was used to guide this study [25], with the population being nurses, the intervention being wearable devices, the control being usual care, and the outcomes being health outcomes (including physical and psychosocial).

### Data Sources and Search Strategy

We searched the following electronic databases from inception until July 2022: PubMed, Embase, CINAHL, Web of Science, IEEE Explore, and AS&T. We consulted an academic-affiliated librarian for the literature search. In addition, we will conduct citation tracking for all eligible studies to highlight articles potentially missed by our search strategy. Textbox 1 displays the specific search and MeSH terms used to identify articles during the search with 2 of the databases. Outcome terms were not included in the search strategy to ensure the search was as inclusive as possible, given the exploratory nature of this review.

**Search Strategy for PubMed and CINAHL.**

**PubMed:**

(wearable* OR “wearable technology” OR “wearable technologies” OR “wearable device” OR “wearable devices” OR “Wearable Electronic Devices”[MeSH] OR “wearable electronic device” OR “wearable electronic devices” OR “Fitness Trackers”[MeSH] OR fitbit* OR smartwatch* OR “apple watch” OR garmin OR actigraph* OR “Actigraphy”[MeSH] OR (“eye-based” AND wearable) OR PolarH10 OR “wearable sensor” OR “wearable sensors” OR (posture AND monitor*) OR (wearable AND monitor*) OR “Wearable Electronic Devices”[MeSH] OR “activity tracker” OR “activity trackers” OR “fitness tracker” OR “fitness trackers” OR “google glass” OR “Smart Glasses”[MeSH] OR “smart glasses” OR smartglass* OR (stress AND wearable*) OR (“blood pressure” AND wearable) OR (sweat AND wearable*) OR (stress AND monitor*) OR (“blood pressure” AND monitor*) OR (sweat AND monitor*) OR (“blood pressure” AND sensor*) OR (sweat AND sensor) OR (stress AND sensor)) AND (Nurse* OR “Nurses”[MeSH])

**CINAHL:**

(wearable* OR “wearable technolog*” OR “wearable device*” OR fitbit* OR “apple watch” OR garmin OR actigraph* OR smartwatch* OR (“eye-based” AND wearable) OR PolarH10 OR “wearable sensor” OR “wearable sensor*” OR (posture AND monitor*) OR “wearable electronic device*” OR (wearable AND monitor*) OR activity tracker*” OR “fitness tracker*” OR “google glass” OR “smart glass*” OR smartglass* OR (MH “Fitness Trackers”) OR (MH “Wearable Sensors”) OR (MH “Actigraphy”) OR (stress AND wearable*) OR (“blood pressure” AND wearable*) OR (sweat AND wearable*) OR (“blood pressure” AND monitor*) OR (sweat AND monitor*) OR (stress AND monitor) OR (“blood pressure” AND sensor*) OR (sweat AND sensor*) OR (stress AND sensor*) ) AND ( nurse* OR (MH “Nurses”) )

### Study Selection and Screening

To be included in this review, studies must meet the following four inclusion criteria: (1) nurses must be in the population studied (>80% of the sample), (2) a wearable device must be included (which the nurses have attached to their clothing, worn as a device anchored directly to their body, or attached directly to their skin, and that has embedded sensors with wireless mobility), (3) there must be a health outcome studied, including physical and psychosocial outcomes, and (4) the study has an observational or interventional design (prospective and retrospective). Studies are excluded if they meet 1 or more of the following exclusion criteria: (1) they do not report on health outcomes, (2) they are specific to nursing students, (3) they use wearable devices with patients, (4) they are not published in English, (5) they are protocols, (6) they are reviews, or (7) they are located only in the gray literature.

Titles and abstracts were imported into Covidence software, where citations will be screened and duplicates removed. Title, abstract, and full-text screening will be conducted independently and in duplicate by 4 reviewers. Cohen κ statistics will be reported to evaluate the interreviewer agreement at different stages. Inclusion and exclusion criteria guide the full-text screening. Cases of disagreement between reviewers regarding study inclusion will be resolved through discussion.

### Data Extraction and Analysis

Four reviewers will extract data independently and in duplicate. We will collect citation information, study design and location, recruitment data, sample information, wearable device data, outcome data, and biometric cross-reporting data using an author-developed data extraction form (Table 1). Given the nonsystematic nature of this review, statistical pooling of inferential estimates will not be conducted. We will report measures of frequency and central tendency to describe sample and study characteristics.

**Table 1:** Data extraction form.

Citation information	Study design and location	Recruitment data	Sample information	Wearable device data	Outcome data	Biometric cross-reporting
First author (year), title	Type of study design, single versus multisite, and type of site	Inclusion and exclusion criteria, time frame, location of data collection	Total sample size, gender and age data, and country	Type and use of wearable device and measures used in reporting	Primary and secondary health outcomes, assessment time points, and key findings	Reporting of biometric wearable sensor data with nurse activities

## Results

Search strategies were developed, and data were extracted from 6 databases in July 2022. After the removal of duplicates, we collected 8603 studies for title and abstract screening. Three independent reviewers conducted the title and abstract review, and after resolving conflicts, there were 277 full-text articles to review. The initial title and abstract review had an overall agreement value of 95.7%, resulting in a moderate Cohen κ of 0.43 for interrater reliability [26]. After the full-text articles are reviewed independently for study eligibility by 4 reviewers, data will be extracted. Additional data may be located after we check the eligible article references. All extracted data will be reported using narrative and tabular formats. We will organize the reported data in accordance with 4 key factors: the type of wearable devices, the wearable measures reported, the health outcomes reported, and the methodology used. Considering the size and depth of this review, we will not report on the feasibility or acceptance of wearable devices in nursing. Results are expected to be available in the fall of 2023.

## Discussion

This integrative review aims to carefully examine studies available in the global literature that have used wearable devices to improve health among nurses. The goal is to evaluate and inform on the measurement of health outcomes obtained via wearable devices among nurses. These studies will include the results of both descriptive and correlational and interventional studies that tested a wearable device to assess the effect on the nurses’ health. Given the importance of physical and psychological health for nurses, we are examining both facets of health. Synthesized results from this review will inform multiple stakeholders, including the nursing workforce, health system administrators, wearable device developers, health insurance companies, policy makers, and mobile health researchers.

As wearable devices continue to evolve rapidly and improve, becoming easier to use while providing richer data sources, it is pertinent that nursing and those supporting their health consider its use and value [27,28]. Using wearable devices for assessing, informing, and providing the information needed to develop healthier behaviors should positively impact the nurses' health and well-being. This should consequently positively affect health care systems that employ nurses, especially in light of nursing labor shortages. Further, the downstream impact of healthy nurses can improve the care and outcomes of the patients they care for in inpatient, outpatient, and community settings.

This work can inform existing movements focused on improving nurses’ health; for example, *Healthy Nurse, Healthy Nation*, a program designed by the American Nurses Association Enterprise [29]. The overarching goal of this program, which has existed in the United States since 2017, is to improve nurses’ health in the nation. The 6 domains they focus on are activity, rest, nutrition, quality of life, mental health, and safety. Longitudinal data have been collected on hospital workers, including nurses, where several of these domains have been studied, including collecting data on physical and mental health markers, demonstrating how data can be collected over time in nurses [30]. In addition, several domains, including physical activity and rest, have been studied with nurse shift workers using commercially available sensors, where they found poorer health outcomes in night shift nurses [31]. For the safety domain, wearable devices are already known to assess these areas with other populations (eg, military and construction workers) [32,33].

Using biometric screening and tracking of occupational activities performed by the nurse provides an opportunity to assess which activities may contribute to poor physical outcomes for the nurse [34]. In addition, predictive analytics can provide context for mitigating those occupational activities that put nurses at risk for adverse health consequences [27]. However, obtaining data from biometric monitoring devices is a field that continues to evolve, and methodological specificities of tools used to obtain biometric data must be carefully reported [35].

Nurses comprise over half of the global health care workforce, and the nursing care they provide is critical for the global population's health [3]. Understanding existing work that has been done to date with the use of wearable devices and focused on nurses’ health has the potential to inform how to improve the health of the nursing workforce. This integrative review will provide synthesized data to inform nurses and other stakeholders about the extent of wearable work done with nurses and provide direction for future research.

## Abbreviations

PICO: population, intervention, control, and outcomes
PRISMA: Preferred Reporting Items for Systematic Reviews and Meta-Analyses
PRISMA-P: Preferred Reporting Items for Systematic Reviews and Meta-Analyses Protocols

## Data Availability

The research team will make available all synthesized data within the text of the manuscript or supplemental files. The data used will already be published and available.
